# Dietary Patterns of Korean Adults and the Prevalence of Metabolic Syndrome: A Cross-Sectional Study

**DOI:** 10.1371/journal.pone.0111593

**Published:** 2014-11-03

**Authors:** Hae Dong Woo, Aesun Shin, Jeongseon Kim

**Affiliations:** 1 Molecular Epidemiology Branch, National Cancer Center, Goyang-si, Korea; 2 Department of Preventive Medicine, Seoul National University College of Medicine, Seoul, Republic of Korea; University of Pittsburgh Medical Center, United States of America

## Abstract

The prevalence of metabolic syndrome has been increasing in Korea and has been associated with dietary habits. The aim of our study was to identify the relationship between dietary patterns and the prevalence of metabolic syndrome. Using a validated food frequency questionnaire, we employed a cross-sectional design to assess the dietary intake of 1257 Korean adults aged 31 to 70 years. To determine the participants’ dietary patterns, we considered 37 predefined food groups in principal components analysis. Metabolic syndrome was defined according to the National Cholesterol Education Program Adult Treatment Panel III. The abdominal obesity criterion was modified using Asian guidelines. Prevalence ratios and 95% confidence intervals for the metabolic syndrome were calculated across the quartiles of dietary pattern scores using log binomial regression models. The covariates used in the model were age, sex, total energy intake, tobacco intake, alcohol consumption, and physical activity. The prevalence of metabolic syndrome was 19.8% in men and 14.1% in women. The PCA identified three distinct dietary patterns: the ‘traditional’ pattern, the ‘meat’ pattern, and the ‘snack’ pattern. There was an association of increasing waist circumference and body mass index with increasing score in the meat dietary pattern. The multivariate-adjusted prevalence ratio of metabolic syndrome for the highest quartile of the meat pattern in comparison with the lowest quartile was 1.47 (95% CI: 1.00–2.15, p for trend = 0.016). A positive association between the prevalence of metabolic syndrome and the dietary pattern score was found only for men with the meat dietary pattern (2.15, 95% CI: 1.10–4.21, p for trend = 0.005). The traditional pattern and the snack pattern were not associated with an increased prevalence of metabolic syndrome. The meat dietary pattern was associated with a higher prevalence of metabolic syndrome in Korean male adults.

## Introduction

Metabolic syndrome is associated with an increased risk of developing type 2 diabetes [1] and cardiovascular disease, as well as general mortality [2], [3]. According to the National Health and Nutrition Examination Survey (NHANES), using the revised National Cholesterol Education Program/Adult Treatment Panel III (ATP III) definition, the age-adjusted prevalence of metabolic syndrome in the US adult Americans significantly increased from 29.2% between 1988 and 1994 to 34.2% between 1999 and 2006 [4]. The age-adjusted prevalence of metabolic syndrome was 13.7% between 2000 and 2001, and prevalence of metabolic syndrome was 26.7% between 2007 and 2008, using ATP III criteria modified for the Asia-Pacific subjects in China [5], [6]. Based on the Korean National Health and Nutrition Examination Survey (KNHANES), using the ATP III criteria from the Asia-Pacific region for central obesity, the age-adjusted prevalence of metabolic syndrome in the Korean population increased from 24.9% in 1998 to 31.3% in 2007 [7]. Trends in prevalence of diabetes in Asian countries have been increased considerably [8], although no significant change has been observed in recent years [9]. Metabolic syndrome risk factors might be closely related to diabetes and cardiovascular disease. Thus their potential causative factors need to be explored.

The risk of metabolic syndrome is known to be associated with dietary intake [10]–[12]. Analysis of dietary patterns could account for the inter-related dietary factors that are potentially important for the development of metabolic syndrome [13]. The dietary patterns of people in developing countries have changed as a result of modernization, which might contribute to an increased risk of obesity and metabolic syndrome [14], [15]. Moreover, migration studies have shown that western dietary patterns lead to an accumulation of fat [16], [17], which may contribute to the development of metabolic syndrome.

In previous studies, Western pattern characterized by high intakes of protein, processed foods, and refined grains was positively associated with metabolic syndrome, whereas healthy dietary pattern characterized by high intakes of fruits, vegetables and dairy was inversely associated with metabolic syndrome [11], [12], [18]–[21]. Recently, several studies were conducted to determine the association between dietary patterns and the prevalence of metabolic syndrome among Koreans [22]–[24]. The traditional Korean meal, which is low in fat and contains a large portion of vegetables, has been considered a healthy diet [14], [25]. However, the traditional Korean dietary pattern was not associated with a lower prevalence of metabolic syndrome, and findings regarding the relationship between dietary pattern and metabolic syndrome have been inconsistent [22]–[24]. The association between dietary patterns and the metabolic syndrome has not been fully identified in the Korean population. Thus, the purpose of this study is to determine the association between various dietary patterns and the prevalence of metabolic syndrome in Korea.

## Methods

### Study population

**Figure 1:**
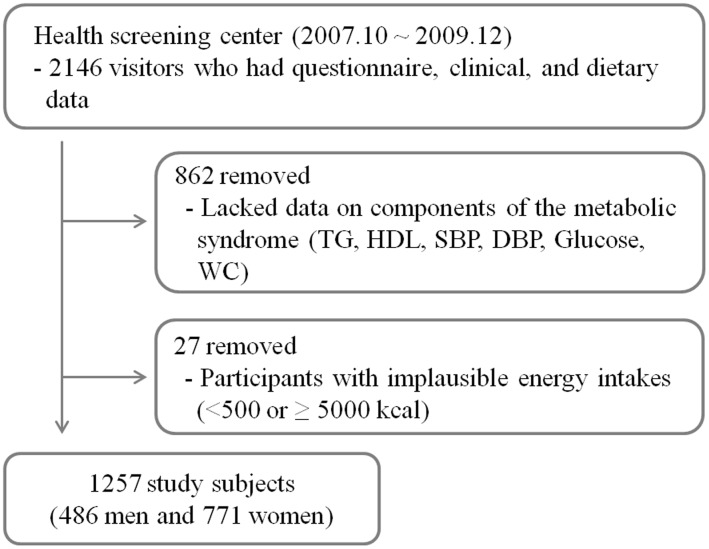
Flow chart of study selection process.

We performed a cross-sectional study of participants who underwent health screening examinations at the Center for Cancer Prevention and Detection at the National Cancer Center in South Korea between October 2007 and December 2009. Visitors are National Health Insurance beneficiaries and those who have all data for survey question including medical history, clinical test result, and dietary consumption data were recruited (n = 2146). No one was excluded due to other diseases such as diabetes, coronary heart disease, stroke, or cancer. We excluded 862 subjects whose medical records lacked data regarding metabolic syndrome components. There was no significant difference in BMI between participants with missing metabolic syndrome components and participants with complete data. Participants with implausible energy intake values (<500 or ≥5000 kcal, n = 27) were excluded. The remaining 1257 adults (486 men and 771 women), ranging between 31 and 70 years old, were used in our analysis (Figure 1). Each participant was provided with an informed consent form according to the procedures approved by the institutional review board of the National Cancer Center. Written informed consent was obtained from all participants.

### Data collection

The participants completed a self-administered questionnaire, which asked about each participant’s demographics, lifestyle, medical history, and diet. Self-reported physical activity was evaluated using an International Physical Activity Questionnaire (IPAQ) short form [26]. The total metabolic equivalent (MET-minutes/week) was a combined score that was calculated by multiplying the frequency, duration, and intensity of physical activity. The dietary intake was assessed using a validated food frequency questionnaire (FFQ) [27]. The participants were asked about their average frequency of intake and portion size of specific foods during the previous year of 103 types of food. Three portion sizes and 9 categories of frequency were specified on the FFQ. The average daily nutrient intake was measured by summing up the intake of associated nutrient content per 100 g for each of the 103 foods. The foods listed in the FFQ were categorized into 37 different food groups, each of which was determined according to the food’s nutrient profile and its culinary use (Table S1).

### Metabolic syndrome definition

Metabolic syndrome was defined according to the National Cholesterol Education Program Adult Treatment Panel III (NCEP-ATP III) [28]. The abdominal obesity criterion was modified using Asian guidelines [29]. Under these definitions, a person has metabolic syndrome if that person exhibits three or more of the following conditions: 1) triglycerides ≥150 mg/dL; 2) HDL cholesterol <40 mg/dL in men or <50 mg/dL in women; 3) systolic blood pressure (BP) ≥130 mmHg, diastolic blood pressure (BP) ≥85 mmHg, or drug treatment for hypertension; 4) fasting glucose ≥110 mg/dL or drug treatment for elevated glucose levels; and 5) waist circumference ≥90 cm in men or ≥80 cm in women.

### Statistical analysis

The general characteristics in the group with metabolic syndrome and the group without metabolic syndrome were compared using a Student t-test for continuous variables and a chi-square test for categorical variables. Principal-components analysis (PROC FACTOR) was used to extract the participants’ dietary patterns using 37 predefined food groups. We used a varimax rotation to enhance the interpretability of the factors that were analyzed. We determined how many factors to retain after evaluating the eigenvalue, scree test, and interpretability. The dietary patterns were named according to the highest factor driving the food groups for each dietary factor. Each dietary pattern’s factor score was categorized by quartile for further analysis. The trend test was performed to analyze the associations between each of the dietary patterns and each of the components of metabolic syndrome using a general linear model with adjustments for confounding factors. Regression analysis in log-log scale of each dietary pattern score and the components of metabolic syndrome was performed to compare with trends across quartiles. Prevalence ratios (PRs) and 95% confidence intervals (CIs) for the metabolic syndrome were calculated across the quartiles of dietary pattern scores using log binomial regression models. The lowest quartile of each dietary pattern was used as the reference. The trend test was performed to analyze the associations between each of dietary pattern score (continuous) and the prevalence of metabolic syndrome using Wald test. PRs and 95% CIs for the metabolic syndrome were also calculated stratified for sex. Model 1 was adjusted according to age, sex (for total), and total energy intake. Model 2 was further adjusted for age, sex (for total), total energy intake, tobacco intake, alcohol consumption, and physical activity. We performed the statistical analysis using SAS version 9.3 (SAS Institute Inc, Cary, NC). All P values were two-tailed (α = 0.05).

## Results

The general characteristics of the study participants are reported in Table 1. The overall prevalence of metabolic syndrome was 16.3%, and the prevalence was significantly higher in men (19.8%) than in women (14.1%). Compared to people who did not have metabolic syndrome, patients who had metabolic syndrome were older (p<0.001) and had a higher BMI (p<0.001). The separate components of metabolic syndrome were significantly different (metabolic syndrome positive vs. negative, Mean (SD): 137.2 (13.1) vs. 124.0 (13.6) for systolic BP, 83.7 (9.5) vs. 75.5 (10.0) for diastolic BP, 105.4 (26.1) vs. 90.7 (16.5) for fasting glucose, 46.9 (8.9) vs. 60.6 (13.9) for HDL cholesterol, 201.6 (81.8) vs. 102.4 (61.2) for triglycerides), depending on whether metabolic syndrome was present or absent (p<0.001).

**Table 1:** General characteristics of the study population, and comparison of individuals with and without metabolic syndrome.

	Total	MS (+)	MS (−)	p value
N (%)	1257	205 (16.3)	1052 (83.7)	
Age (y)	51.7 (9.2)	55.9 (9.2)†	50.8 (9.0)	<0.001
Sex, n (%)				
Male	486 (38.7)	96 (19.8)	390 (80.3)	0.009
Female	771 (61.3)	109 (14.1)	662 (85.9)	
Smoking status, n (%)				
Never	774 (63.6)	114 (57.9)	660 (64.7)	0.164
Former	288 (23.7)	52 (26.4)	236 (23.1)	
Current	155 (12.7)	31 (15.7)	124 (12.2)	
Alcohol consumption, n (%)				
Never	472 (38.9)	78 (39.8)	394 (38.8)	0.943
Former	102 (8.4)	17 (8.7)	85 (8.4)	
Current	638 (52.6)	101 (51.5)	537 (52.9)	
Total energy intake (kcal)	2175.7 (857.3)	2282.6 (915.4)	2154.9 (844.4)	0.051
Saturated fatty acid (g/d)	7.9 (4.8)	8.2 (5.0)	7.9 (4.7)	0.987‡
Carbohydrates (g/d)	387.1 (163.1)	401.2 (165.3)	384.4 (162.6)	<0.001‡
Fiber (g/d)	34.3 (32.6)	37.4 (35.7)	33.7 (32.0)	0.563‡
Sodium (mg/d)	3207.0 (1675.9)	3623.8 (2198.5)	3125.8 (1542.0)	<0.001‡
Total vegetables (g/d)	177.5 (130.1)	195.9 (149.2)	173.9 (125.8)	0.114‡
Fruits (g/d)	221.5 (249.7)	210.3 (219.7)	223.6 (255.2)	0.193‡
BMI (Kg/m^2^)	23.8 (3.0)	26.8 (2.6)	23.2 (2.7)	<0.001
Waist circumference (cm)	79.0 (8.8)	87.5 (6.7)	77.4 (8.2)	<0.001
Systolic BP (mmHg)	126.2 (14.4)	137.2 (13.1)	124.0 (13.6)	<0.001
Diastolic BP (mmHg)	76.8 (10.4)	83.7 (9.5)	75.5 (10.0)	<0.001
Fasting glucose (mg/dL)	93.1 (19.2)	105.4 (26.1)	90.7 (16.5)	<0.001
HDL cholesterol (mg/dL)	58.3 (14.1)	46.9 (8.9)	60.6 (13.9)	<0.001
Triglycerides (mg/dL)	118.6 (74.6)	201.6 (81.8)	102.4 (61.2)	<0.001
Physical activity (MET-min/wk)	2934.2 (2946.5)	2885.2 (2810.7)	2943.7 (2973.5)	0.795

MS (−), absence of metabolic syndrome; MS (+), presence of metabolic syndrome; MET, metabolic equivalent.

† Numbers are Mean (SD), unless otherwise stated.

‡ Adjusted for total energy intake.

The PCA identified three major dietary patterns, and their factor-loading scores are shown in Table 2. The ‘traditional’ dietary pattern included high intakes of condiments, green/yellow vegetables, light-colored vegetables, tubers, clams, tofu/soymilk, and seaweed; the ‘meat’ dietary pattern included high intakes of red meat, red meat byproducts, other seafood, and high-fat red meat; and the ‘snack’ pattern included high intakes of cake/pizza, snacks, and bread. Three patterns explained 31.9% of the total variance.

**Table 2:** Factor loadings of the dietary patterns derived from principal components analysis with orthogonal rotation.

	Traditional pattern	Meat pattern	Snack pattern
Condiments	0.78		0.26
Green/yellow vegetables	0.74		
Light colored vegetables	0.71	0.40	
Tubers	0.67		0.32
Clams	0.63	0.22	0.26
Tofu, soymilk	0.61		0.22
Seaweeds	0.60		
Bonefish	0.54		
Kimchi	0.49		
Lean fish	0.46	0.37	
Mushrooms	0.42	0.36	
Fruits	0.40		
Nuts	0.37		
Legumes	0.29		
Yogurt	0.27		
Eggs	0.27		0.28
Pickled vegetables	0.24		
Milk	0.20		
Red meat	0.23	0.79	
Red meat by-products		0.74	
Other seafood	0.25	0.67	
High-fat red meat		0.60	
Oil		0.50	0.20
Salted fermented seafood		0.44	
Noodles		0.43	
Poultry		0.43	
Fatty fish		0.37	0.29
Carbonated beverages		0.36	0.27
Dairy products		0.30	0.25
Cakes, pizza			0.81
Snacks			0.68
Bread			0.60
Processed meats		0.29	0.50
Sweets		0.28	0.36
Rice cake			0.23
Coffee, tea		0.20	
Grains			
Variance explained (%)	18.8	7.5	5.6

Factor loadings with absolute values <0.2 are not presented.

Table 3 shows the associations between each dietary pattern and the components of metabolic syndrome, including BMI, and general characteristics according to quartiles of each dietary pattern score. The traditional and snack patterns showed no relationships with any of the components of metabolic syndrome. Increasing scores in the meat dietary pattern were associated with elevated waist circumference, BMI, triglycerides, blood pressure, and low concentrations of HDL cholesterol (p<0.05). The traditional pattern score increased with an increment of age and physical activity of each quartile, but with decrease in percentage of men and current drinker. The meat pattern score increased with both increment of age and percentage of men and current drinker, but no difference was observed in physical activity. The snack pattern score increased with a decrement of age, but no differences were observed in percentage of men and current drinker, and physical activity.

**Table 3:** The association between dietary patterns and the components of metabolic syndrome and BMI*.

	Quartile of dietary pattern score					
	Q 1	Q 2	Q 3	Q 4	p for trend†	p value‡
Traditional pattern (n)	285	263	275	260		
Waist circumference (cm)	78.1 (9.3)	78.6 (8.9)	78.3 (8.8)	78.3 (7.8)	0.790	0.841
Triglyceride (mg/dL)	115.0 (78.3)	116.8 (75.2)	113.5 (64.2)	117.9 (78.3)	0.450	0.063
HDL cholesterol (mg/dL)	58.7 (14.6)	58.7 (14.5)	58.9 (13.9)	59.4 (13. 7)	0.410	0.582
Diastolic BP (mmHg)	76.1 (10.6)	76. 7 (10.5)	76.1 (10.2)	76.7 (10.4)	0.407	0.134
Systolic BP (mmHg)	124.7 (14.4)	126.1 (14.1)	124.5 (13.8)	126.3 (15.0)	0.189	0.593
Fasting glucose (mg/dL)	89.2 (12.9)	91.5 (14.0)	91.0 (15.7)	93.3 (25.9)	0.538	0.177
Body Mass Index (kg/m^2^)	23.4 (3.0)	23.6 (3.0)	23.8 (3.0)	23.6 (2.7)	0.960	0.976
Age (yr)	48.9 (8.7)	51.6 (9.4)	51.9 (9.3)	54.2 (8.9)	<0.001	
Male, n (%)	138 (28.4)	135 (27.8)	107 (22.0)	106 (21.8)	0.001	
Current drinker, n (%)	174 (27.3)	173 (27.1)	153 (24.0)	138 (21.6)	<0.001	
Physical activity (MET-min/wk)	2636.5 (2812.3)	2895.4 (3047.1)	3065.5 (3040.0)	3138.7 (2868.2)	<0.001	
Total energy intake	1691.3 (707.7)	2052.4 (753.0)	2303.0 (786.8)	2654.5 (873.1)	<0.001	<0.001
Carbohydrates§	397.0 (2.4)	395.1 (2.3)	387.2 (2.3)	369.2 (2.4)	<0.001	<0.001
Fat§	31.3 (0.8)	31.4 (0.8)	33.6 (0.8)	39.2 (0.8)	<0.001	<0.001
Protein§	71.2 (1.1)	73.4 (1.0)	79.1 (1.0)	89.8 (1.1)	<0.001	<0.001
Fiber§	31.3 (1.6)	30.4 (1.6)	35.6 (1.6)	40.0 (1.6)	<0.001	<0.001
Sodium§	2226.3 (69.5)	2779.8 (66.6)	3236.7 (66.6)	4580.9 (69.3)	<0.001	<0.001
Saturated fat§	7.2 (0.2)	7.6 (0.2)	7.9 (0.2)	9.0 (0.2)	<0.001	<0.001
Meat pattern (n)	273	267	266	277		
Waist circumference (cm)	76.8 (7.7)	77.8 (8.9)	78.0 (8.9)	80.6 (8.9)	<0.001	<0.001
Triglyceride (mg/dL)	108.6 (67.7)	109.9 (69.2)	110.2 (63.8)	133.7 (89.7)	0.018	0.005
HDL cholesterol (mg/dL)	60.5 (14.7)	59.3 (13.8)	58.9 (13.7)	56.9 (14.4)	0.032	0.067
Diastolic BP (mmHg)	75.7 (10.3)	75.2 (10.2)	76.7 (11.0)	77.8 (10.0)	0.034	0.854
Systolic BP (mmHg)	124.4 (13.8)	124.9 (14.4)	125.4 (15.1)	126.7 (14.1)	0.018	0.073
Fasting glucose (mg/dL)	91.9 (20.1)	91.7 (12.7)	88.9 (10.2)	92.3 (24.0)	0.954	0.050
Body Mass Index (kg/m^2^)	23.1 (2.6)	23.6 (3.0)	23.4 (2.8)	24.3 (3.1)	<0.001	<0.001
Age (yr)	54.7 (8.5)	51.8 (8.9)	50.9 (9.3)	49.3 (9.4)	<0.001	
Male, n (%)	86 (17.7)	111 (22.8)	128 (26.3)	161 (33.1)	<0.001	
Current drinker, n (%)	123 (19.3)	146 (22.9)	166 (26.0)	203 (31.8)	<0.001	
Physical activity (MET-min/wk)	2950.1 (2951.2)	2963.5 (2991.0)	2880.4 (2785.7)	2942.8 (3064.2)	0.466	
Total energy intake	2116.8 (923.5)	2004.6 (805.9)	2059.3 (723.9)	2520.7 (868.2)	<0.001	<0.001
Carbohydrates§	408.9 (2.0)	398.6 (2.0)	387.1 (2.0)	353.9 (2.1)	<0.001	<0.001
Fat§	26.2 (0.7)	29.4 (0.7)	33.8 (0.7)	46.1 (0.7)	<0.001	<0.001
Protein§	71.6 (1.1)	76.2 (1.1)	78.5 (1.1)	87.2 (1.1)	<0.001	<0.001
Fiber§	33.4 (1.6)	37.7 (1.6)	34.5 (1.6)	31.7 (1.6)	0.259	0.629
Sodium§	2857.5 (77.3)	2908.1 (77.9)	3226.3 (77.6)	3834.2 (78.9)	<0.001	<0.001
Saturated fat§	6.5 (0.2)	6.6 (0.2)	7.9 (0.2)	10.6 (0.2)	<0.001	<0.001
Snack pattern (n)	257	273	275	278		
Waist circumference (cm)	79.8 (8.4)	78.0 (8.4)	78.1 (9.0)	77.2 (8.7)	0.051	0.006
Triglyceride (mg/dL)	117.1 (75.0)	120.5 (76.1)	112.8 (71.7)	112.7 (73.8)	0.833	0.413
HDL cholesterol (mg/dL)	58.6 (13.7)	58.8 (13.7)	58.0 (13.7)	60.2 (15.4)	0.827	0.936
Diastolic BP (mmHg)	77.9 (10.4)	76.2 (10.1)	75.9 (11.1)	75.6 (9.8)	0.124	0.134
Systolic BP (mmHg)	127.2 (13.6)	125.1 (14.0)	124.7 (15.1)	124.6 (14.6)	0.353	0.364
Fasting glucose (mg/dL)	93.2 (14.5)	90.6 (11.8)	90.7 (22.0)	90.4 (20.5)	0.410	0.343
Body Mass Index (kg/m^2^)	24.0 (2.8)	23.4 (2.7)	23.6 (3.1)	23.5 (3.0)	0.506	0.115
Age (yr)	53.9 (8.8)	52.5 (8.9)	51.3 (9.4)	48.9 (9.2)	<0.001	
Male, n (%)	132 (27.2)	124 (25.5)	116 (23.9)	114 (23.5)	0.111	
Current drinker, n (%)	159 (24.9)	167 (26.2)	148 (23.2)	164 (25.7)	0.965	
Physical activity (MET-min/wk)	2964.9 (2944.4)	2797.5 (2876.0)	3095.5 (2946.4)	2879.3 (3023.0)	0.427	
Total energy intake	2087.9 (836.7)	2009.1 (860.9)	2113.8 (761.9)	2491.1 (886.0)	<0.001	<0.001
Carbohydrates§	395.6 (2.2)	397.8 (2.2)	387.1 (2.2)	368.1 (2.3)	<0.001	<0.001
Fat§	29.4 (0.8)	29.4 (0.8)	34.1 (0.8)	42.5 (0.8)	<0.001	<0.001
Protein§	78.5 (1.1)	76.6 (1.1)	77.8 (1.1)	80.6 (1.1)	0.134	0.229
Fiber§	34.4 (1.6)	37.1 (1.6)	33.9 (1.6)	31.9 (1.6)	0.141	0.093
Sodium§	3340.2 (79.7)	2963.4 (80.1)	3123.5 (79.9)	3399.6 (81.0)	0.349	0.238
Saturated fat§	7.0 (0.2)	7.0 (0.2)	8.2 (0.2)	9.5 (0.2)	<0.001	<0.001

*Participants who were taking medication for hypertension and elevated glucose were excluded for the analysis of the components of metabolic syndrome and BMI.

† General linear model with adjustments for age, sex, smoking status, alcohol consumption, total energy intake, and physical activity (log-transformed) for the analysis of the components of metabolic syndrome and BMI, and adjustments for total energy intake for the analysis of nutrients.

‡ Regression analysis in log-log scale with adjustments for age, sex, smoking status, alcohol consumption, total energy intake, and physical activity (log-transformed) for the analysis of the components of metabolic syndrome and BMI, and adjustments for total energy intake for the analysis of nutrients.

§ Least squares means (SE) adjusted for total energy intake.

Numbers are Mean (SD), unless otherwise stated.

The association between the PR of metabolic syndrome and the dietary pattern score variables are shown in Table 4. The score variable of the meat pattern was associated with the prevalence of metabolic syndrome in both models (p for trend = 0.006 and 0.016, respectively). The multivariate-adjusted PR of metabolic syndrome for the highest quartile of the meat pattern in comparison with the lowest quartile was 1.47 (95% CI: 1.00–2.15, p for trend = 0.016). We found no association between the prevalence of metabolic syndrome and either the snack pattern score variables or the traditional pattern score variables. The association between the dietary pattern scores and the prevalence of metabolic syndrome was analyzed after stratifying by sex. A positive association between the prevalence of metabolic syndrome and the dietary pattern score was found only for men with the meat dietary pattern (multivariate-adjusted PR of the highest group compared with the lowest group: 2.15, 95% CI: 1.10–4.21, p for trend = 0.005).

**Table 4:** PRs and 95% CIs of metabolic syndrome by quartiles of dietary patterns.

	Dietary pattern	Quartiles of dietary pattern scores			p for trend*
		Q2	Q3	Q4	
Total	Traditional (n)	314	314	315	
(1257)	Model 1†	1.04 (0.71–1.53)§	1.01 (0.69–1.50)	1.02 (0.69–1.52)	0.408
	Model 2‡	1.01 (0.68–1.51)	1.17 (0.79–1.75)	1.08 (0.71–1.63)	0.330
	Meat (n)	313	314	315	
	Model 1†	1.16 (0.81–1.67)	1.23 (0.86–1.76)	1.40 (0.98–1.99)	0.006
	Model 2‡	1.23 (0.84–1.81)	1.33 (0.91–1.94)	1.47 (1.00–2.15)	0.016
	Snack (n)	314	313	315	
	Model 1†	0.77 (0.55–1.07)	0.82 (0.59–1.15)	0.86 (0.62–1.21)	0.249
	Model 2‡	0.79 (0.56–1.13)	0.90 (0.64–1.28)	0.93 (0.65–1.32)	0.421
Men	Traditional (n)	135	107	106	
(n = 486)	Model 1†	0.99 (0.59–1.66)	1.05 (0.61–1.83)	1.12 (0.64–1.94)	0.195
	Model 2‡	0.96 (0.56–1.64)	1.26 (0.72–2.18)	1.18 (0.66–2.10)	0.129
	Meat (n)	111	128	161	
	Model 1†	1.23 (0.67–2.27)	1.27 (0.70–2.30)	1.68 (0.94–2.98)	0.005
	Model 2‡	1.64 (0.82–3.27)	1.70 (0.86–3.34)	2.15 (1.10–4.21)	0.005
	Snack (n)	124	116	114	
	Model 1†	0.57 (0.34–0.96)	0.87 (0.55–1.37)	0.80 (0.49–1.30)	0.314
	Model 2‡	0.53 (0.31–0.94)	0.91 (0.57–1.45)	0.80 (0.49–1.31)	0.335
Women	Traditional (n)	179	207	209	
(n = 771)	Model 1†	1.18 (0.67–2.08)	0.99 (0.56–1.76)	0.98 (0.55–1.74)	0.932
	Model 2‡	1.16 (0.63–2.12)	1.14 (0.63–2.07)	1.07 (0.58–1.97)	0.978
	Meat (n)	202	186	154	
	Model 1†	1.21 (0.76–1.92)	1.29 (0.82–2.02)	1.15 (0.71–1.87)	0.248
	Model 2‡	1.18 (0.73–1.91)	1.26 (0.78–2.02)	1.14 (0.68–1.92)	0.455
	Snack (n)	190	197	201	
	Model 1†	1.03 (0.66–1.61)	0.82 (0.50–1.33)	1.01 (0.63–1.61)	0.685
	Model 2‡	1.11 (0.69–1.80)	0.89 (0.53–1.51)	1.11 (0.66–1.85)	0.830

PR: prevalence ratio, CI: confidence interval.

*Trend test were performed by Wald test using continuous variables of each pattern score (log-transformed).

† Adjusted for age, sex (for total) and total energy intake.

‡ Adjusted for age, sex (for total), total energy intake, smoking status, alcohol consumption, and physical activity (log-transformed).

§ PR (95% CI), compared with quartile 1 as a reference.

## Discussion

The present study derived three dietary patterns in the Korean adult population: the ‘traditional’ pattern, the ‘meat’ pattern, and the ‘snack’ pattern. We found that the meat dietary pattern score was positively associated with the prevalence of metabolic syndrome especially in men, whereas the traditional pattern and the snack pattern were not associated with metabolic syndrome.

Because Korean meals often include mixed soups or multiple side dishes comprising various vegetables, tubers, tofu, and seaweed with condiments, Factor 1 was labeled as the traditional dietary pattern. The traditional Korean meal is low in fat and contains a large portion of vegetables, which can be considered a healthy diet. However, the traditional pattern was not associated with a lower prevalence of metabolic syndrome in our results or in previous studies [22]–[24]. HDL cholesterol was inversely associated with the traditional pattern score derived from 16 food groups in women [22]. The traditional pattern score was inversely associated, although not statistically significant, with HDL cholesterol, and positively associated with a prevalence of metabolic syndrome in Hong et al. [23]. Another study that used cluster analysis showed that HDL cholesterol was lower in people with the traditional pattern compared with the those of both meats and alcohols pattern and Korean healthy pattern [24]. Grain, especially refined grain was highly correlated with the traditional pattern in the three above Korean studies. Thus it seems that the negative association between HDL cholesterol and the Korean traditional food pattern was substantially affected by high intakes of carbohydrate. HDL cholesterol was negatively related with carbohydrate [30] and glycemic index [31]. However, grain had very low factor loading for the traditional pattern in our study. Traditional Korean foods are usually cooked with condiments that contain high levels of salt. Highest factor loading in the traditional pattern was condiments, and the pattern score of the traditional pattern was highly correlated with sodium intake in our study (r = 0.72, p<0.001). This may have led to a lack of an association between the traditional Korean dietary pattern and the prevalence of metabolic syndrome in our study. Therefore, the Korean traditional pattern is not generally associated with the prevalence of metabolic syndrome, but high sodium intakes could increase the risk of metabolic syndrome.

The western diet, which has high factor loadings for red meat and processed meat, was positively related to metabolic syndrome [11], [12], [21], [32], whereas meat intake was not associated with metabolic syndrome in French adults [33]. The food groups with high factor loadings in the meat dietary pattern in our study were different from those with high factor loadings in the western dietary pattern and in the meat dietary pattern investigated in the previous studies. Both the western dietary pattern and the meat pattern generally had a high factor loading for processed meat, which is responsible for many of the adverse effects that are characteristic of the meat dietary pattern. Poultry, which is usually found in healthy dietary patterns, was instead characteristic of the meat pattern in our study. The poultry eaten in Korea was mostly cooked by boiling in 1990. However, the majority of poultry eaten changed to fried chicken in 1998 according to KNHANES. Consequently, poultry has become a major source of saturated fat. Despite discrepancies between each of the food groups, our results suggest that the meat pattern is associated with the prevalence of metabolic syndrome in male adults. This association has been consistently observed in the results from previous studies of the western and meat dietary patterns. The positive association was only found in the male group in our study, suggesting that the meat dietary pattern among Korean adults might increase the prevalence of metabolic syndrome in males to a greater extent than in females.

Several possible mechanisms may explain the detrimental effect of the meat pattern in the human body. First, meat is a major source of total fat intake, particularly saturated fat, and the consumption of saturated fat has been associated with plasma lipoprotein levels [34] and higher blood pressure levels [35]. In a group of individuals of Japanese ancestry, red meat consumption was associated with a higher risk of developing metabolic syndrome among men. However, this association was no longer significant after making adjustments for saturated fatty acids [36]. Thus, the prevalence of metabolic syndrome in the meat pattern was analyzed with further adjustments for saturated fatty acids in our study. The prevalence ratio of metabolic syndrome in the highest quartile compared with the lowest quartile was attenuated (data not shown). Second, meat intake is related to the deposition of iron, particularly heme-iron. Metabolic syndrome subjects had a significantly higher prevalence of iron overload than control subjects [37], and high ferritin concentrations were positively associated with the prevalence of metabolic syndrome and with insulin resistance [38], [39]. It was suggested that high iron contents of red meat might be related with higher prevalence of metabolic syndrome [40], [41]. Meat intake, especially processed meat, was associated with increased risk of coronary heart disease and diabetes in meta-analysis [42]. Exact mechanism is not explained clearly, but iron overload increase oxidative stress due to its catalytic properties [43], resulting insulin resistance and decreased insulin secretion [44], [45]. Additionally, the meat dietary pattern may be closely related to a high consumption of alcohol in our study, which may have increased the prevalence of metabolic syndrome in these individuals. Food groups with high factor loadings in the meat pattern were often consumed with alcohol. In previous studies that included alcohol as a food for dietary pattern analysis, a ‘meats and alcohols’ pattern was derived, suggesting that meat and alcohol consumption are highly correlated in Korean diets [23], [24]. Heavy drinking was positively associated with metabolic syndrome and its components [46], [47], and both alcohol consumption and a meat dietary pattern were associated with an increased prevalence of metabolic syndrome [21]. The percentage of current drinkers was higher in the highest quartile of meat pattern in our study, especially in men. Although it was adjusted for in the analysis, alcohol consumption may partly affect the prevalence of metabolic syndrome in the meat pattern. Drinking habits might explain the sex difference in the meat pattern as well, as men are more likely to drink heavily than women. Another explanation for the gender difference is body iron stores. It was suggested that a lower incidence of heart diseases in women, especially in premenopausal women, might be related to lower body iron stores [48], [49]. A significant association between iron-related genes and type 2 diabetes was observed in men but not in women [50].

The snack pattern was not associated with the prevalence of metabolic syndrome and its components. Women who had a fiber bread pattern had a lower prevalence of metabolic syndrome and higher insulin sensitivity, while a white bread pattern was positively associated with metabolic syndrome and lowered insulin sensitivity [51], [52]. Whole grain consumption was inversely associated with type 2 diabetes [53], a higher waist-to-hip ratio, LDL-cholesterol and fasting insulin [52]. Thus the type of grain consumed by individuals with a snack pattern may affect the prevalence of metabolic syndrome. However, the snack pattern score was only slightly correlated with carbohydrate and fiber in our study. The snack pattern score increased with a decrement of age, and waist circumference, which was highly correlated with age, was inversely associated with the snack pattern score. Thus the trend of decreasing age with increasing the snack pattern score, although age was adjusted for in the analysis, may affect the prevalence of metabolic syndrome in the snack pattern. The meat pattern score also increased with a decrement of age, but it still positively associated with the prevalence of metabolic syndrome. It suggests that the association between the meat pattern and the prevalence of metabolic syndrome is strong.

Our study has several limitations. Because this is a cross-sectional study, there is a chance that dietary intake was affected by an individual’s health status, which makes it difficult to find a true association between dietary intake and metabolic syndrome. In addition, we cannot exclude of the possibility of measurement errors of study variables and residual confounding. Thus associations identified should be interpreted in caution. The prevalence of metabolic syndrome in our study was lower than that in previous reports that analyzed the KNHANES data [7]. The study participants may have had a healthier lifestyle, as they volunteered for the health screening examinations, therefore leading to the lower prevalence of metabolic syndrome. The three dietary patterns derived from PCA analysis explained about 32% of the total variation; thus the derived patterns might not explain all Korean dietary patterns thoroughly.

In conclusion, the meat dietary pattern, which was characterized by a high consumption of red meat, red meat byproducts, and high-fat red meat, was associated with an increased prevalence of metabolic syndrome in Korean male adults.

## Supporting Information

Table S1 **Food lists of 37 food groups.**
(DOCX)
