# Supplementary material for: Dietary Patterns of Korean Adults and the Prevalence of Metabolic Syndrome: A Cross-Sectional Study
Source: PLoS One. 2014 Nov 3;9(11):e111593. doi: 10.1371/journal.pone.0111593 (PMC4218781; doi:10.1371/journal.pone.0111593)
Supplement: Table S1 — Food lists of 37 food groups. (DOCX) [file pone.0111593.s001.docx]

Table S1. Food lists of 37 food groups

| Food groups | Lists |
| --- | --- |
| Green/yellow vegetables | Carrot, spinach, sesame leaf, lettuce, zucchini, water dropwort, sweet potato tips, lactuca, pepper, pepper leaf, leaf beet, shepherd's purse, broccoli, radish leaf, tomato, celery, crown daisy, Korean leek, mugwort |
| Light colored vegetables | Bean sprout, cabbage, radish root, onion, bellflower, bracken, deodeok (mountain herb), garlic, green onion, lotus root |
| Kimchi | Kimchi |
| Pickled vegetables | Garlic, garlic stem, radish root |
| Tubers | Potato, sweet potato, starch, dangmyeon (starch noodle) |
| Legumes | Pea, black soybeans |
| Mushrooms | Mushrooms |
| Fruits | Apple, banana, pear, orange, grape, persimmon, mandarin, strawberry, peach, watermelon, oriental melon, muskmelon, processed fruits |
| Nuts | Acorn jelly, peanut, almond, pine nut, sesame seed |
| Grains | Rice, millet, flour, barly, cereal, adlay |
| Noodles | Noodles |
| Bread | Bread |
| Rice cake | Tteok (Korean rice cake) |
| Cakes, pizza | Cakes and pizza |
| Sweets | Chocolate, honey, candy, sugar, starch syrup |
| Snacks | Biscuit, cookie, cracker, snacks |
| Tofu, soymilk | Tofu, soymilk |
| Milk | Milk |
| Yogurt | Liquid, curd yogurt |
| Dairy products | Ice-cream, sherbet, cheese |
| Red meat | Beef, pork |
| Processed meats | Luncheon meat, bacon, sausages, ham, burgers |
| Poultry | Chicken |
| High-fat red meat | Beef rib, pork rib, pork belly, fat pork |
| Red meat by-products | Red meat by-products |
| Eggs | Eggs |
| Oil | Butter, margarine, sesame oil, soy oil, beef tallow |
| Condiments | Soy source, mustard, red pepper powder, red pepper paste, sesame salt, soy paste, salt, vinegar, pepper powder |
| Coffee, tea | Coffee and tea |
| Carbonated beverages | Carbonated beverages |
| Fatty fish | Mackerel, mackerel pike, Spanish mackerel, bass, tuna, eel |
| Lean fish | Hairtail, pollack, yellow corvine, halibut, sea bream |
| Bonefish | Anchovy |
| Clams | Clams, mussels, oysters, and scallops |
| Other seafood | Crab, shrimp, octopus, squid, processed fish cake |
| Salted fermented seafood | Salted fermented seafood |
| seaweeds | Gim (dried laver), miyeok (sea mustard), dashima (kelp) |
